# Occurrence of Dumping Syndrome After Esophageal Cancer Surgery: Systematic Review and Meta-analysis

**DOI:** 10.1245/s10434-024-15881-x

**Published:** 2024-07-27

**Authors:** Yuan Lin, Hejie Wang, Yaxin Qu, Zhiqiang Liu, Pernilla Lagergren, Shao-Hua Xie

**Affiliations:** 1 School of Public Health, Fujian Medical University, Fuzhou, China; 2 Department of Molecular Medicine and Surgery, Karolinska Institutet, Stockholm, Sweden; 3 Department of Surgery and Cancer, Imperial College London, London, UK; 4 Institute of Population Medicine, Fujian Medial University, Fuzhou, China; 5 Key Laboratory of Ministry of Education for Gastrointestinal Cancer, Fujian Medical University, Fuzhou, China

**Keywords:** Dumping symptoms, Esophageal neoplasms, Esophagectomy, Postoperative complications, Systematic review

## Abstract

**Background:**

Dumping syndrome occurs frequently after esophageal cancer surgery, but the reported prevalence varied across previous studies. This systematic review and meta-analysis aimed to clarify the prevalence of dumping syndrome after esophageal cancer surgery, particularly exploring the sources of heterogeneity in previous studies.

**Methods:**

A comprehensive literature search was conducted in PubMed, MEDLINE, Web of Science, Embase, and the Cochrane Library databases, supplemented by hand-search of reference lists, through March 2023. Random-effects meta-analysis estimated the average prevalence of dumping syndrome after esophageal cancer surgery. Heterogeneity across studies was examined by the *I*^2^ statistic and Cochran’s *Q* test.

**Results:**

Among the 2949 articles retrieved from the databases, 16 articles (15 cohort studies and 1 randomized controlled trial) met the inclusion criteria. The prevalence of dumping syndrome ranged 0–74% in these studies, showing high heterogeneity (*I*^2^ = 99%, *P* < 0.01), with the pooled prevalence of 27% (95% confidence interval [CI] 14–39%). The pooled prevalence in the three studies using specialized questionnaires was 67% (95% CI 60–73%), with reduced heterogeneity (*I*^2^ = 43%, *P* = 0.17). The prevalence also varied by year of publication, study population, and length and completeness of follow-up.

**Conclusions:**

Our findings revealed that dumping syndrome is common after esophageal cancer surgery. The varying prevalence across previous studies was probably owing to differences in measurement of dumping syndrome. Using specific patient reported outcome questionnaires is recommended for future investigations on dumping syndrome after esophageal cancer surgery.

**Supplementary Information:**

The online version contains supplementary material available at 10.1245/s10434-024-15881-x.

Esophageal cancer is the seventh most common type of cancer and sixth leading cause of cancer-related deaths worldwide.^1^ It has a poor prognosis after diagnosis, with an overall 5-year survival of less than 20% in most populations globally. Esophagectomy, often combined with chemo- and/or radiotherapy, is the main curative intent treatment for esophageal cancer.^2^ Despite improvements in surgical procedures and postoperative supports in recent years, postoperative complications remain common in esophageal cancer patients and has been linked to a poor long-term survival.^3^

Dumping syndrome is a unique type of postoperative complication in esophageal cancer patients. Dumping syndrome occurs when the gastric-emptying mechanism is altered, which is caused by changes in the anatomy of the gastrointestinal tract and disruption of its innervation. This leads to rapid passage of food into the small intestine, causing symptoms, such as syncope, palpitations, abdominal cramps, and nausea in the patient.^4^ Dumping syndrome presents as early or late dumping with distinct pathophysiological and symptomatic features that correspond to the time interval after a meal.^5^ Early dumping symptoms occur within 1 hour after a meal, which is characterized by abdominal cramps, diarrhea, palpitations, and nausea. Late dumping symptoms occur 1 to 3 h after a meal, which usually presents with symptoms of hypoglycemia.^6^ Patients can experience either early dumping symptoms, late dumping symptoms, or both. Dumping syndrome symptoms are frequently debilitating and emotionally distressing, leading to a significant decline in quality of life. In addition, these symptoms are related to decreased food intake, which can result in consequential weight loss.^7^

Previous studies have reported varying prevalence of dumping syndrome after esophageal cancer surgery.^8^ However, no quantitative synthesis of data or examination of heterogeneity was conducted. Therefore, this updated systematic review and meta-analysis aimed at assessing the occurrence of dumping syndrome after surgery in esophageal cancer patients and exploring the sources of heterogeneity across previous studies.

## Methods

This study was conducted and reported in accordance with the Preferred Reporting Items for Systematic reviews and Meta-Analyses (PRISMA) and the Meta-analysis Of Observational Studies in Epidemiology (MOOSE) guidelines.^9,10^

### Literature Search

We systematically searched the MEDLINE, Embase, PubMed, Web of Science, and Cochrane Library databases to capture potentially eligible articles from the date of inception until March 12, 2023. In brief, a combination of key words for “esophageal cancer,” “esophagectomy or surgery or operation,” and “dumping syndrome” were searched in title or abstract. The specific search strategies are illustrated in detail in the *Supplementary Materials*. To identify additional studies that might have been missed in search in electric databases, we also reviewed the reference lists of the included studies and relevant review articles.

### Selection Criteria

Titles and abstracts of the identified articles were reviewed in the first round of screening, and full text reports were further referred to for studies considered as potentially relevant, after which eligibility was assessed according to the predetermined inclusion and exclusion criteria. We included original and independent cohort studies and clinical trials, with access to full text, followed up to investigate the occurrence of dumping syndrome in esophageal cancer patients who underwent curative intent surgery. Articles meeting any of the following were excluded: (1) being written in languages other than English; (2) reviews, comments, protocols, editorials, letters, case reports, or animal studies; (3) duplicate publications; and 4) studies without data sufficient for calculating a proportion and its 95% confidence interval (CI). If multiple studies from the same cohort were identified, the most recent study was selected for analysis. Two authors (Y.L. and H.W.) independently screened each article for eligible studies. Uncertainties were resolved through joint reevaluation and verified by a senior investigator (S.H.X.).

### Study Quality Assessment

We assessed the quality of observational studies in terms of risk of selection bias, information misclassification and confounding, with the help of the Newcastle-Ottawa Scale (NOS) for assessing the quality of nonrandomized studies in meta-analyses.^11^ The NOS scoring system rates the study quality of nonrandomized epidemiological studies on the three major methodological aspects, i.e., risk of selection bias, comparability between groups, and information bias (assessment of exposure or outcome), which are assessed by eight specific items. An overall score ranging from 0 to 9 was given to each original study; higher scores indicated higher study quality. We assessed methodological quality and risk of bias in randomized controlled trials by using the Cochrane tool for assessing risk of bias, version 2 (RoB 2).^12^ The RoB 2 tool assess the quality of randomized controlled trials in terms of the following five sources of bias: whether randomization is appropriate, the effect of assignment and adhering to the intervention, loss to follow-up, misclassification of outcomes, and the analysis and selection of the reported data; the overall risk of bias from an individual study will be judged as “low,” “some concerns,” or “high” risk of bias. The assessment of study quality was processed independently by two authors (Y.L. and H.W.), and any discrepancies were dealt with by discussion or consultation with a senior investigator (S.H.X.).

### Data Extraction

The following data were extracted from the included studies: first author, year of publication, type of study design, country, number of participants, age and gender distribution of participants, length of follow-up, completeness of follow-up, measuring method of dumping syndrome, surgical approach (transthoracic, transhiatal, or minimally invasive esophagectomy), anastomosis approach (cervical or intrathoracic), preoperative neoadjuvant therapy (yes or no), preoperative adjuvant therapy (yes or no), and number of patients with dumping syndrome. Two authors (Y.L. and Y.Q.) separately extracted the data and compared the consistency together; disagreement between the two sets of data was solved by discussion, with involvement of a senior investigator ((S.H.X.) whenever necessary.

### Statistical Analysis

Because of the considerate heterogeneity across individual studies, we conducted random-effects meta-analysis to estimate the pooled prevalence (proportion) of dumping syndrome after esophageal cancer surgery and its 95% CI. Heterogeneity across studies was quantitatively assessed by using the Cochran’s Q test and I^2^ statistic.^13^ We assessed potential publication bias with the help of funnel plots, supplemented by quantitative evaluation by the Begg’s and Egger’s tests.^14,15^ Subgroup analyses were conducted by the following factors to assess the sources of heterogeneity between individual studies: by measuring method of dumping syndrome (using specialized questionnaires, or not), published year (before 2000, or in 2000 or later), study population (Western or Eastern Asian), length of follow-up (≤ 6 months, ≤ 12 months, or > 12 months) and completeness of follow-up (loss to follow-up ≤20%, or >20%). We conducted sensitivity analyses by dropping individual primary studies one by one to examine the fluctuation of the pooled prevalence of dumping syndrome.

All analyses were performed using the software R (version 4.2.3) and the meta package (version 6.2-1).^16^ All statistical tests were two-sided, *P* value < 0.05 was considered to be statistically significant.

## Results

### Literature Search and Study Characteristics

The detailed procedure of identification and selection of articles is presented in Supplementary Fig. S1. The literature search identified a total of 2256 studies after duplicates were removed. Among these, 10 studies fulfilled the eligibility criteria. Six additional studies were identified through reviewing reference lists of relevant articles. Thus, a total of 16 studies, including 15 cohort studies and one randomized clinical trial, were enlisted in this systematic review.^17–32^ These studies included 2240 patients who underwent curative surgery for esophageal cancer. Detailed characteristics of each study are shown in Tables 1 and 2. The overall quality of the 15 cohort studies was rated either 5 or 6 in the NOS scoring system (Supplementary Table S1), and the included randomized trial showed “some concerns” in methodological quality according to the RoB 2 tool (Supplementary Table S2).

**Table 1:** Characteristics of included studies

References	Country	Study period	Male (%)	Age (years)	No. patients	Follow-up (months)	Loss to follow-up	Assessment of dumping syndrome	No. events (%)
*Cohort studies*									
Mannell et al.^17^	South Africa	Unspecified	66.70%	Range 35–60	15	1.25–48	0.0%	“Clinical assessment"	2 (13.0%)
King et al.^18^	United States	1980–1982	81.00%	Mean 60.6 (range 33–91)	95	2–74.4	5.3%	Medical records	5 (5.0%)
Collard et al.^19^	Belgium	1984–1987	76.50%	Range 23–66	17	36–76	0.0%	Questionnaire	3 (6.0%)
Wang et al.^20^	China	1974-1984	96.50%	Mean 61.6 (range 29–82)	76	>12	79.4%	Questionnaire	10 (13.2%)
Kuwano et al.^21^	Japan	1986–1990	86.00%	Mean 59.86	50	60	2-year 28.0%, 3-year 60.0%, 4-year 80.0%, 5-year 92.0%	Unspecified	6 (12.0%)
Orringer et al.^22^	United States	1976–unspecified	79.60%	Average 63 (range 29–92)	377	1–181	9.6%	Unspecified	182 (48.0%)
Finley et al.^23^	Canada	1980-1994	79.30%	Mean 64 (range 16–86)	169	3	5.1%	Postprandial lightheadedness or diarrhea	10 (6.0%)
McLarty et al.^24^	United States	1972–1990	75.70%	Median 62 (range 30–81)	107	>60	32.7%	Mail survey	53 (50.0%)
Aghajanzadeh et al.^25^	Iran	1993–2003	69.80%	Mean 48 (range 22–75)	192	12–48	20.0%	Self-administered health questionnaire	79 (46.0%)
Antonoff et al.^26^	United States	2007-2012	84.60%	Mean 61.8 ± SD 0.6	140	>12	6-month, 6.8%, 12-month 43.0%, >12 months 52.2%	Patient reported symptoms	25 (17.9%)
Anandavadivelan et al.^27^	Sweden	2013–2018	86.70%	Average 66 ± SD 8.6)	188	0–18	1-year 15.3%, 1.5- year 33.1%	Sigstad’s score and the Arts dumping questionnaire	129 (69.0%)
Klevebro et al.^28^	United States	1995–2017	80.70%	Median 66.2 (range 30-90)	159	3–277	7.0%	Dumping Symptom Rating Scale	97 (61.0%)
Yoshida et al.^29^	Japan	2008–2019	84.00%	Mean 67.6 ± SD 9.1	300	1–60	52.3%	Unpecified	7 (2.3%)
Bennett et al.^30^	Ireland	2017–2019	84.00%	Mean 63.3	35	6–12	6-month 12.0%, 12-month 50.7%	Sigstad’s score	26 (74.3%)
Chen et al.^31^	China	2020–2021	85.00%	Mean 62.6 ± SD 7.1	20	–	0.0%	Unspecified	0 (0.0%)
*Randomized controlled trial*									
Li et al.^32^	China	2015–2017	86.7%	Range 18–75	300	1–30	0.0%	Unspecified	0 (0.0%)

*SD* Standard deviation

**Table 2:** Surgical approaches and other treatments in the included studies

References	Pyloric procedure	Surgical approach	Graft position	Conduit	Site of anastomosis	Neoadjuvant therapy	Adjuvant therapy
*Cohort studies*							
Mannell et al.^17^	–	TTE	–	G	CE (53%), IT (47%)	–	–
King et al.^18^	PP (56%), PM (39%)	TTE	PM	G	IT	–	–
Collard et al.^19^	PP (100%)	TTE (65%), THE (18%), Other (17%)	PM (65%), RS (35%)	G (82%), C (18%)	CE	–	–
Wang et al.^20^	PP (24%)	TTE(93%), THE (7%)	–	G	CE	–	–
Kuwano et al.^21^	PP (100%)	TTE	PM (34%), RS (48%), SC (18%)	G (96%), C (4%)	CE (66%), IT (34%)	–	–
Orringer et al.^22^	PM (routine)	TTE (3%), THE (97%)	PM (96%), RS (3%), Other (1%)	G (95%), C (4%), Other (1%)	CE	Yes	–
Finley et al.^23^	PP (6.4%), PM (80.0%)	TTE (26%), THE (74%)	–	G	CE (93%), IT (7%)	Yes	–
McLarty et al.^24^	PP (34%), PM (49%)	THE ( 85%), TTE (4 %), Other (11%)	–	G (93%), C (3%), SB (4%)	CE (19%), IT (81%)	Yes	–
Aghajanzadeh et al.^25^	PP (22%), PM (47%)	TTE (26%), THE (74%)	–	G (80%), C (15%), SB (5%)	CE (90%), IT (10%)	–	Yes
Antonoff et al.^26^	PP (12.3%), PM (54.9%), FB (2.7%), FB+BI (15.0%)	TTE (56%), THE (44%)	–	G (98.6%)	–	–	–
Anandavadivelan et al.^27^	–	MIE (30%), TTE (30%), Other (30%)	–	–	–	Yes	–
Klevebro et al.^28^	PP (2.3%)	TTE (88%), THE (2%), Other (10%)	–	–	IT (55%), CE (45%)	Yes	–
Yoshida et al.^29^	–	TTE	PM (84%), RS (16%)	G	CE	Yes	–
Bennett et al.^30^	PP	THE (27%), Other (73%)	–	C	–	Yes	–
Chen et al.^31^	–	MIE	–	–	–	–	–
*Randomized controlled trial*							
Li et al.^32^	–	THE	–	SB	–	–	–

*BI* botulinum injection; *C* colon; *CE* cervical; *E* esophagus; *FB* finger bougie; *G* gastric; *IT* intrathoracic; *MIE* minimally invasive esophagectomy; *PM* (for graft position) posterior mediastinum; *PM* (for pyloric procedure) pyloromyotomy; *PP* pyloroplasty; *RS* retrosternal; *SB* small bowel; *SC* subcutaneous; *THE* transhiatal esophagectomy; *TTE* transthoracic esophagectomy

### Meta-analysis

**Fig. 1:**
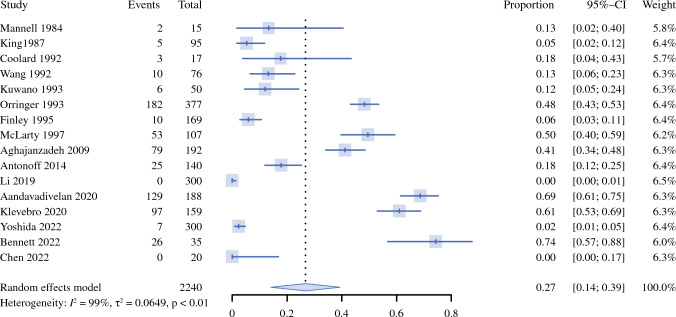
Forest plots for meta-analysis of 16 studies on the prevalence of dumping syndrome after esophageal cancer surgery

The prevalence of dumping syndrome ranged 0–74% in the 16 individual studies, and the meta-analysis estimated a pooled prevalence of 27% (95% CI 14–39%) (Fig. 1). The *I*^2^ statistics and Cochran's Q test showed high heterogeneity across the included studies (*I*^2^ = 99%, *P* < 0.01 in Cochran’s *Q* test). Possible publication bias was revealed by visual inspection of funnel plots, i.e., seemingly more reports of relatively low proportion of dumping syndrome in small-size studies (Supplementary Fig. S2); the Begg’s and Egger’s tests showed *P* values of 0.5285 and 0.0010, respectively.

### Subgroup Analyses

**Fig. 2:**
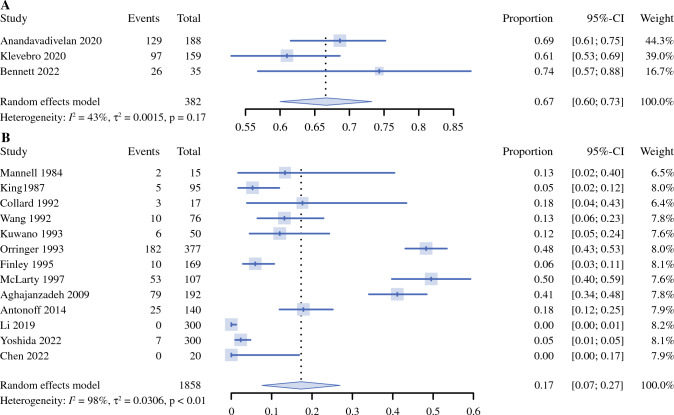
Forest plots for meta-analyses on the prevalence of dumping syndrome after esophageal cancer surgery for in studies using **A** specialized questionnaires **B** and not using

The detailed results of subgroup analyses by measuring method of dumping syndrome, year of publication, study population, and length of follow-up are presented in Table 3, Fig. 2, and Supplementary Fig. S3. Only three of 16 studies used specialized questionnaires for measuring dumping syndrome.^27,28,30^ The pooled prevalence of dumping syndrome in these three studies using specialized questionnaires was higher than that generated from the remaining studies (67%, 95% CI 60–73% vs. 17%, 95% CI 7–27%), with reduced heterogeneity across studies (*I*^2^ = 43%, *P* < 0.01 in Cochran’s *Q* test) (Table 3; Fig. 2).

**Table 3:** Subgroup meta-analyses on prevalence of dumping syndrome after esophageal cancer surgery

Subgroups	No. studies	Pooled prevalence (95% confidence interval)	*P*_Heterogeneity_	*I*^2^ (%)	Begg’s test		Egger’s test	
					Z	*P*	*t*	*P*
*Use of professional questionnaires*								
Yes	3	0.67 (0.60–0.73)	0.17	43	0.52	0.6015	0.51	0.7021
No	13	0.17 (0.07–0.27)	< 0.01	98	0.49	0.6255	3.25	0.0077
*Year of publication*								
Before 2000	8	0.21 (0.08–0.34)	< 0.01	97	1.48	0.1376	0.64	0.5486
2000 or later	8	0.33 (0.11–0.55)	< 0.01	99	1.73	0.0833	3.64	0.0109
*Study population*								
Western	9	0.39 (0.21–0.56)	< 0.01	99	1.04	0.2971	1.83	0.1107
Eastern Asian	5	0.05 (0.00–0.10)	< 0.01	84	1.47	0.1416	2.68	0.0750
*Length of follow-up (months)*								
≤6	4	0.22 (0.00–0.51)	< 0.01	98	2.04	0.0415	6.10	0.0259
≤12	4	0.41 (0.07–0.75)	< 0.01	98	0.68	0.4969	0.29	0.8006
>12	8	0.27 (0.09–0.44)	< 0.01	97	0.99	0.3223	0.98	0.3644
*Loss to follow-up rate*								
≤20%	9	0.21 (0.06–0.37)	< 0.01	99	0.42	0.6767	2.57	0.0371
>20%	7	0.34 (0.12–0.55)	< 0.01	99	1.05	0.2931	2.66	0.0449

The pooled prevalence of dumping syndrome in meta-analysis of the eight studies published before 2000 ranged was 21% (95% CI 8–34%), whereas the pooled prevalence in the other eight studies in 2000 or later was 33% (95% CI 11–55%) (Table 3; Supplementary Fig. S3). Among the 16 included studies, nine studies were from Western populations,^18,19,22–24,26–28,30^ five studies from Eastern Asian population,^20,21,28,31,32^ one study from South Africa,^17^ and one study was from Iran.^25^ The pooled prevalence of dumping syndrome in studies in Western populations was 39% (95% CI 21–56%) in meta-analysis, which was higher than that in Eastern Asian populations (5%, 95% CI 0–10%) (Table 3; Supplementary Fig. S3). Two of 15 patients in the study in South Africa had dumping syndrome, whereas 79 out of 192 patients in the study in Iran reported dumping syndrome. The pooled prevalence of dumping syndrome varied by length of follow-up, i.e., 22% (95% CI 0–51%) within 6 months, 41% (95% CI 7–75%) within 6–12 months, and 27% (95% CI 9–44%) in 12 months or longer after esophageal cancer surgery (Table 3; Supplementary Fig. S4). Meta-analysis of the nine studies with relatively complete follow-up (loss to follow-up ≤ 20%) estimated a prevalence of 21% (95% CI 6–37%) after esophageal cancer surgery.^17–19,22,23,25,28,31,32^ Of the other seven studies with a loss to follow-up, rates >20% estimated a prevalence of 34% (95% CI 12–55%) (Table 3; Supplementary Fig. S4).^20,21,24,26,27,29,30^ High heterogeneity across studies was indicated in most of the subgroup analyses, and no obvious publication bias was observed except for studies assessing prevalence within 6 months of follow-up (Table 3).

### Sensitivity Analyses

Sensitivity analyses by omitting one individual study at a time showed no substantial changes in the pooled estimates (Supplementary Fig. S5).

## Discussion

This systematic literature review with meta-analysis showed varying prevalence of postoperative dumping syndrome in esophageal cancer patients across previous studies. The pooled prevalence was higher in studies using specialized questionnaires for measuring dumping syndrome with reduced heterogeneity across studies. The prevalence of dumping syndrome also was higher in studies published in 2000 or later than in earlier studies, was higher in Western populations than in Eastern Asian populations, and varied by length and completeness of follow-up.

The relatively high prevalence of dumping syndrome and reduced heterogeneity in studies using specialized questionnaires, indicating that the varying prevalence of dumping syndrome across previous studies may be largely due to differences in measuring method of dumping syndrome. Use of unspecific questionnaire or unstandardized “clinical assessment” might have underestimated the occurrence of dumping syndrome in the remaining studies. Therefore, use of specific symptom-based questionnaires, preferably after external validation, should be encouraged in future investigations on dumping syndrome after esophageal cancer surgery. An international consensus on the diagnosis and management of dumping syndrome in 2020 highlighted the need for uniform diagnosis of dumping syndrome, particularly development and validation of specific patient-reported outcome questionnaires.^33^ Three of the 16 included studies used questionnaires specifically designed for assessing dumping syndrome, i.e., the Sigstad’s score, the Dumping Syndrome Rating Scale by Laurenius, or the Arts dumping questionnaire. These questionnaires cover a range of symptoms including borborygmus, nausea, headaches, and dizziness. The Sigstad’s score and the Dumping Syndrome Rating Scale were initially developed for assessing dumping syndrome after peptic ulcer surgery and gastric bypass surgery, respectively, and have been applied in patients who have undergone other types of gastroesophageal surgery, such as gastric and esophageal cancer surgery and bariatric surgery. The Sigstad’s score is based on a total of 16 symptoms of dumping syndrome to which different scores are given, and a diagnostic index is calculated according to the total score. It has been proposed that a score > 7 indicates dumping syndrome and a score < 4 suggests diagnoses other than dumping syndrome.^34^ However, the diagnostic accuracy of the Sigstad’s score, particularly that for such cutoff points, has not been well validated for patients undergoing cancer surgery. The Dumping Syndrome Rating Scale is a questionnaire based on nine symptoms related to early dumping syndrome: one associated with fluid intake and one related to sweetened drinks intake, generating a summary score. The validity of the Dumping Syndrome Rating Scale has been tested in patients undergoing gastric bypass surgery but remains to be further tested in cancer patients.^35^ The Arts dumping questionnaire assesses the severity of eight symptoms of early syndrome and six symptoms of late dumping syndrome, using a 4-point Likert scale where 0 indicates absent, 1 mild, 2 relevant, and 3 indicates severe. However, the Arts dumping questionnaire has not been formally validated.^7^

Apart from symptom-based diagnosis, a modified oral glucose tolerance test is believed to be useful in the diagnosis of dumping syndrome. Specifically, according to an international consensus, a 3% increase in hematocrit or an 10 bpm increase in pulse rate in the first 30 minutes after glucose intake suggests early dumping syndrome, and hypoglycemia after 2 to 3 h suggests late dumping syndrome.^5,6^ Single plasma glucose measurements can be conducted during postoperative clinical visits; however, it has shown limited diagnostic value for dumping syndrome. Nevertheless, continuous monitoring of glucose levels is potentially beneficial for managing complex cases with dumping syndrome.^6^

Surgical factors, such as surgical approach, also may influence the occurrence of dumping syndrome after esophageal cancer surgery. However, only one study compared the prevalence of dumping syndrome in patients who had undergone surgery by different approaches and showed a slightly higher prevalence after open esophagectomy (63%) than minimally invasive surgery (54%) or hybrid thorascopic/laparoscopic surgery (55%).^27^ Differences in patients’ characteristics may explain the high heterogeneity across studies to some extent, but this has not been specifically explored in previous studies.

The considerably high prevalence of dumping syndrome after esophageal cancer surgery, particularly as reported in studies using specific symptom-based questionnaires, should draw attention from medical personnel and caregivers. Dumping syndrome is mainly associated with the vagus nerve division inevitably involved in esophagectomy to allow radical tumor resection and may not be prevented in most cases. However, increased awareness of dumping syndrome is needed to help patients understand what their dumping symptoms represent and more readily report to healthcare for interventions, and it also is valuable for healthcare workers to know the considerably high frequency of dumping syndrome when planning follow-up appointments, because severe dumping symptoms need more intensive medical follow-up and care support. Various measures often are required before the optimal interventions are found for each individual patient. Modifications with resting after meals, dietary changes with meal sizes, timing and contents (e.g., less high-sugar foods), and medical interventions often are used in various combinations to manage dumping symptoms. Timely recognition of the occurrence of dumping syndrome, particularly in patients at high risk of severe dumping symptoms, would depend on a better understanding of risk factors for dumping symptoms. Therefore, more well-designed studies are needed to identify the risk factors for dumping syndrome after esophageal cancer surgery, explore the physical and functional reasons behind dumping syndrome, and assess strategies preventing its occurrence and improving management of dumping syndrome.

There are several limitations in this systematic review and meta-analysis. First, considering the high heterogeneity across studies as elaborated above, the quantitative synthesize of previous studies, i.e., meta-analysis, should be interpreted with caution and probably for reference only. Second, because of the limited number of included studies, estimates in subgroup analyses were lacking accuracy and stratifications by other factors, e.g., patients’ characteristics was not possible. Third, few studies have provided detailed data on severity or spectrum of specific symptoms, or separated early and late dumping syndrome, for which detailed analysis were not possible. Fourth, the quality of the included studies was fair and has limited the strengths of conclusions to some extent. Finally, we only included articles published in English, although it has been increasingly recognized that restriction to English-language publications has little impact on the conclusions of systematic reviews.^36,37^

## Conclusions

This systematic review and meta-analysis comprehensively synthesizing the existing evidence suggests considerably high prevalence of postoperative dumping syndrome in esophageal cancer patients. Previous studies have shown high heterogeneity, which is probably explained by differences in definition and measuring method of dumping syndrome, surgical factors, and patients’ characteristics. More specifically designed studies using validated assessment tools, including specific patient-reported outcome questionnaires, are warranted to investigate the occurrence and risk factors of dumping syndrome after esophageal cancer surgery in the future.

## Supplementary Information

Below is the link to the electronic supplementary material.

Supplementary file1 (PDF 894 kb)
