# Supplementary material for: Occurrence of Dumping Syndrome After Esophageal Cancer Surgery: Systematic Review and Meta-analysis
Source: Ann Surg Oncol. 2024 Jul 27;32(2):791–800. doi: 10.1245/s10434-024-15881-x (PMC11698775; doi:10.1245/s10434-024-15881-x)
Supplement: Supplementary file 1 — Supplementary file1 (PDF 894 kb) [file 10434_2024_15881_MOESM1_ESM.pdf]

Supplementary Materials

Detailed search strategies 2

Supplementary Table 1. Newcastle-Ottawa Scale quality scores of the 15 cohort studies 4

Supplementary Table 2. Risk of Bias 2 of included randomized controlled trial 5

Supplementary Fig. 1 PRISMA flowchart of literature search and study selection 6

Supplementary Fig. 2 Funnel plots for meta-analysis of prevalence of dumping syndrome after esophageal cancer surgery 7

Supplementary Fig. 3 Forest plots for meta-analyses on the prevalence of dumping syndrome after esophageal cancer surgery by year of publication (A) and study population (B) 8

Supplementary Fig. 4 Forest plots for meta-analyses on the prevalence of dumping syndrome after esophageal cancer surgery by length (A) and completeness (B) of follow-up 9

Supplementary Fig. 5 Sensitivity analysis by dropping one individual study at each time 10

Detailed search strategies

Embase&Medline

Step	Keywords
1	((gastrointestinal tract) or esophageal or oesophageal or esophagus or oesophagus or (upper digestive) or (upper aerodigestive) or (upper gastrointestinal)):ti,ab
2	(tumour or tumor or malignan* or neoplasm* or cancer or carcinoma or adenocarcinoma):ti,ab
3	#1 AND #2
4	((dumping syndrome*) or (postgastrectomy syndrome*) or hypoglyc* or diarrhea or dizziness or sweating or diaphoresis or syncope or perspiration or nausea or palpitation or tremor or confusion or tachycardia or hypotension):ti,ab
5	(esophagectomy or surgery or operation):ti,ab
6	#4 AND #5
7	#3 AND #6

PubMed

Step	Keywords
1	(gastrointestinal tract[Title/Abstract]) OR esophageal[Title/Abstract] OR oesophageal[Title/Abstract] OR esophagus[Title/Abstract] OR oesophagus[Title/Abstract] OR (upper digestive[Title/Abstract]) OR (upper aerodigestive[Title/Abstract]) OR (upper gastrointestinal[Title/Abstract])
2	tumour[Title/Abstract] OR tumor[Title/Abstract] OR malignan*[Title/Abstract] OR neoplasm*[Title/Abstract] OR cancer[Title/Abstract] OR carcinoma[Title/Abstract] OR adenocarcinoma[Title/Abstract]
3	Esophageal Neoplasms[MeSH Terms]
4	(#1 AND #2)OR #3
5	Dumping Syndrome[MeSH Terms]
6	(dumping syndrome*[Title/Abstract]) OR (postgastrectomy syndrome*[Title/Abstract]) OR hypoglyc*[Title/Abstract] OR diarrhea[Title/Abstract] OR dizziness[Title/Abstract] OR sweating[Title/Abstract] OR diaphoresis[Title/Abstract] OR syncope[Title/Abstract] OR perspiration[Title/Abstract] OR nausea[Title/Abstract] OR palpitation[Title/Abstract] OR tremor[Title/Abstract] OR confusion[Title/Abstract] OR tachycardia[Title/Abstract] OR hypotension[Title/Abstract]
7	esophagectomy[Title/Abstract] OR surgery[Title/Abstract] OR operation[Title/Abstract]

8	(#5 OR #6) AND #7
9	#4 AND #8

Web of Science

Step	Keywords
1	TS=((gastrointestinal tract) or esophageal or oesophageal or esophagus or oesophagus or (upper digestive) or (upper aerodigestive) or (upper gastrointestinal))
2	TS=(tumour or tumor or malignan* or neoplasm* or cancer or carcinoma or adenocarcinoma)
3	#1 AND #2
4	TS=((dumping syndrome*) or (postgastrectomy syndrome*) or hypoglyc* or diarrhea or dizziness or sweating or diaphoresis or syncope or perspiration or nausea or palpitation or tremor or confusion or tachycardia or hypotension)
5	TS=(esophagectomy or surgery or operation)
6	#4 AND #5
7	#3 AND #6

Cochrane Library

Step	Keywords
1	((gastrointestinal tract) or esophageal or oesophageal or esophagus or oesophagus or (upper digestive) or (upper aerodigestive) or (upper gastrointestinal)):ti,ab,kw
2	(tumour or tumor or malignan* or neoplasm* or cancer or carcinoma or adenocarcinoma):ti,ab,kw
3	MeSH descriptor: [Esophageal Neoplasms] this term only
4	(#1 AND #2)OR #3
5	MeSH descriptor: [Dumping Syndrome] this term only
6	((dumping syndrome*) or (postgastrectomy syndrome*) or hypoglyc* or diarrhea or dizziness or sweating or diaphoresis or syncope or perspiration or nausea or palpitation or tremor or confusion or tachycardia or hypotension):ti,ab,kw
7	(esophagectomy or surgery or operation):ti,ab,kw
8	(#5 OR #6) AND #7
9	#4 AND #8

Supplementary Table 1. Newcastle-Ottawa Scale quality scores of the 15 cohort studies

First author and year	Selection	Comparability	Outcome	Sum
Mannell 1984	3	0	2	5
King 1987	3	0	3	6
Wang 1992	3	0	2	5
Collard 1992	3	0	2	5
Kuwano 1993	3	0	2	5
Orringer 1993	3	0	2	5
Finley 1995	3	0	2	5
McLarty 1997	3	0	2	5
Aghajanzadeh 2009	3	0	2	5
Antonoff 2014	3	2	1	6
Anandavadivelan 2020	4	1	1	6
Klevebro 2020	4	0	2	6
Yoshida 2020	3	0	2	5
Bennett 2022	3	0	3	6
Chen 2022	3	0	2	5

Supplementary Table 2. Risk of Bias 2 of included randomized controlled trial

First author and year	Bias arising from the randomization process	Bias due to deviations from intended interventions	Bias due to missing outcome data	Bias in measurement of the outcome	Bias in selection of the reported result	Overall bias
Li 2019	Some concerns	Low	Low	Low	Low	Some concerns

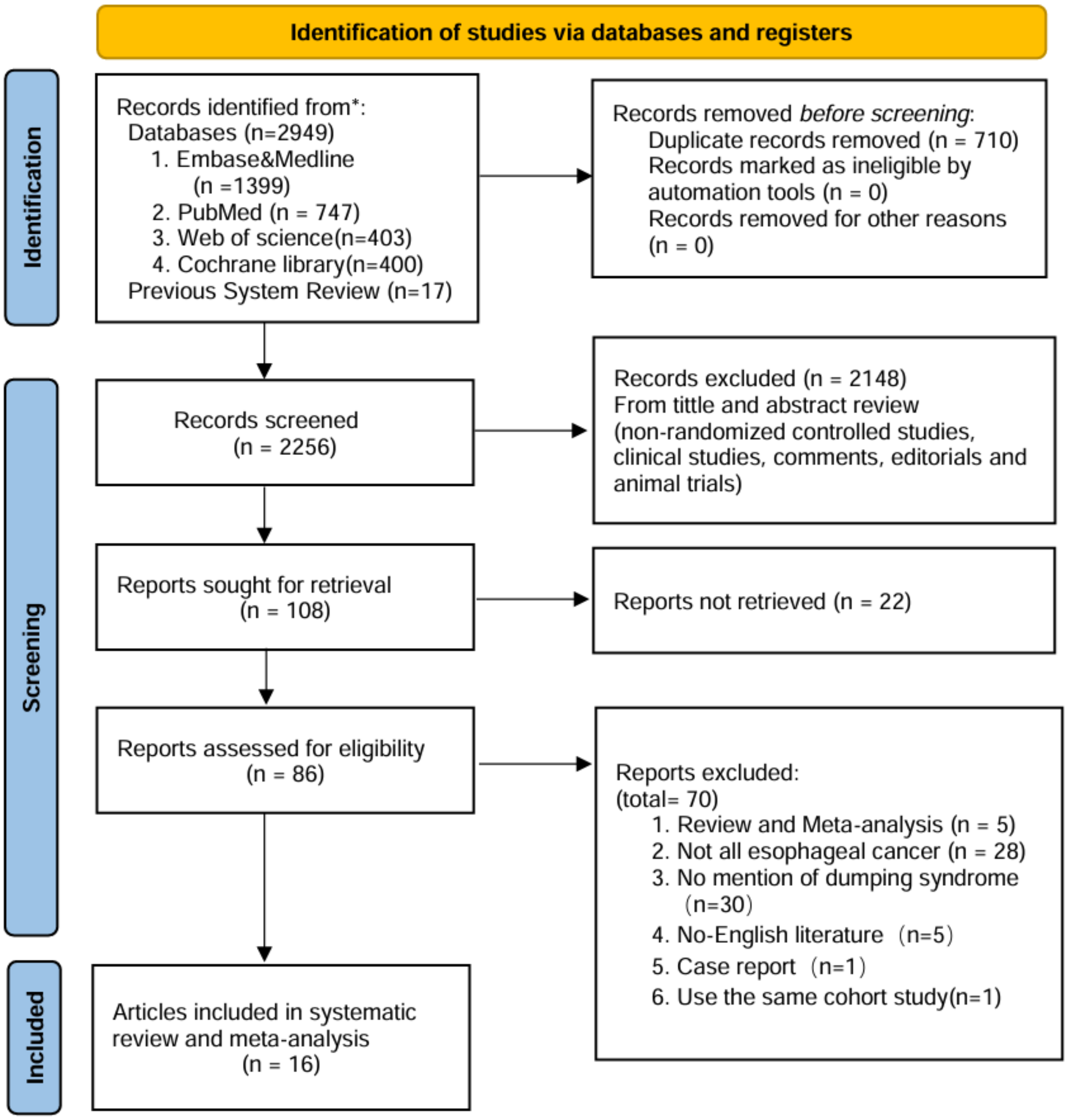

Supplementary Fig. 1 PRISMA flowchart of literature search and study selection

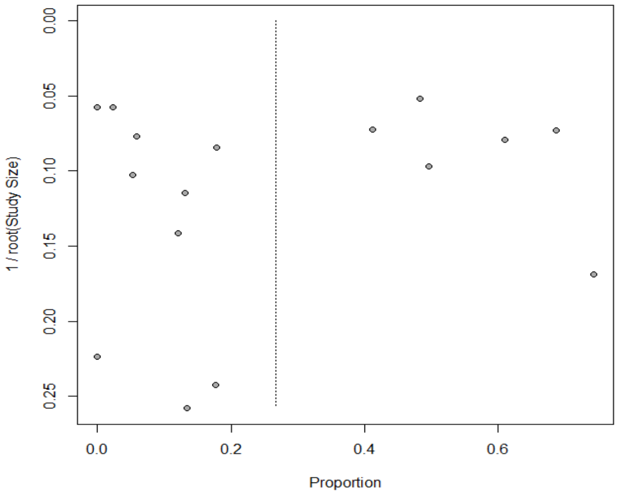

Supplementary Fig. 2 Funnel plots for meta-analysis of prevalence of dumping syndrome after esophageal cancer surgery

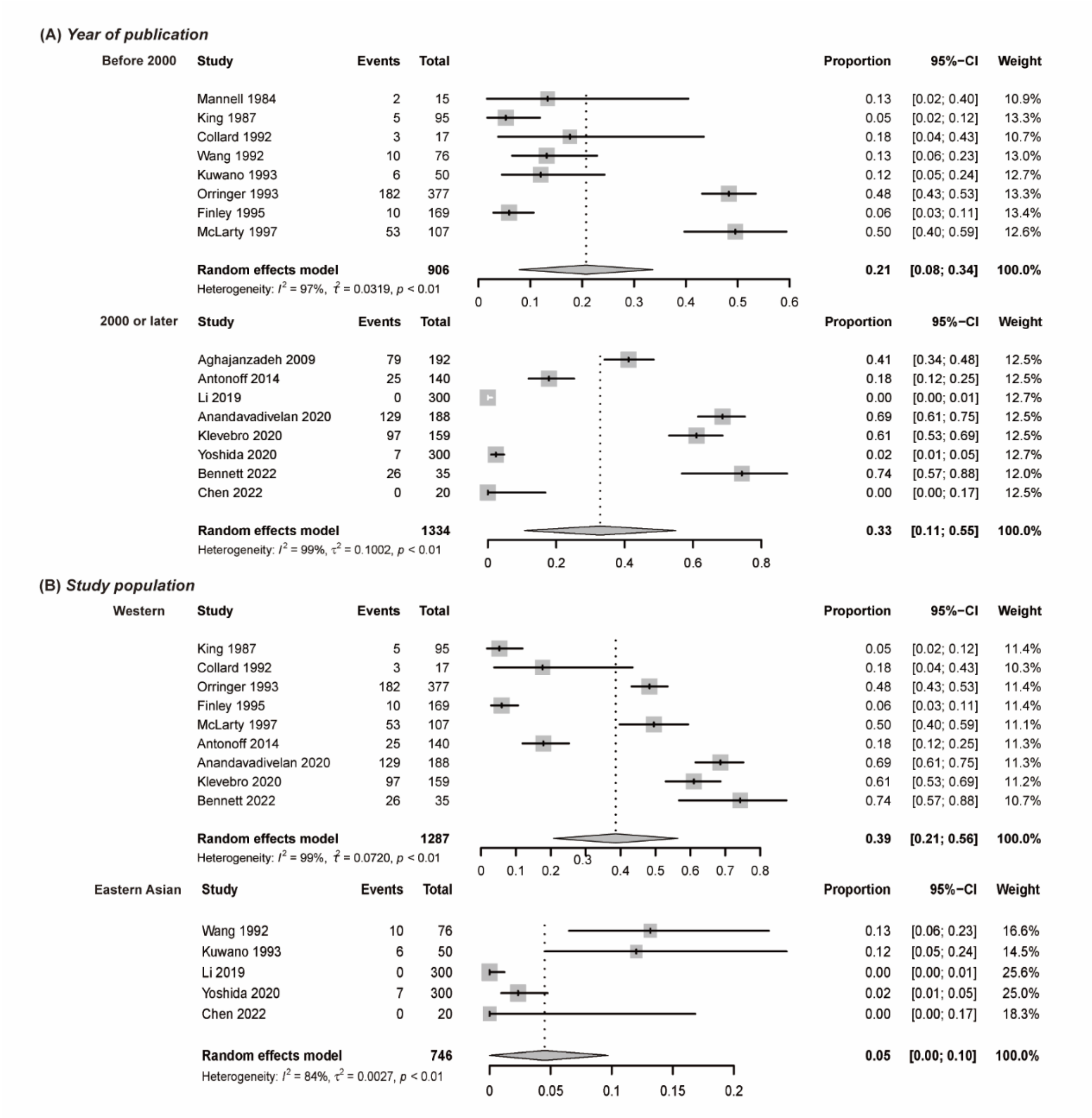

Supplementary Fig. 3 Forest plots for meta-analyses on the prevalence of dumping syndrome after esophageal cancer surgery by year of publication (A) and study population (B)

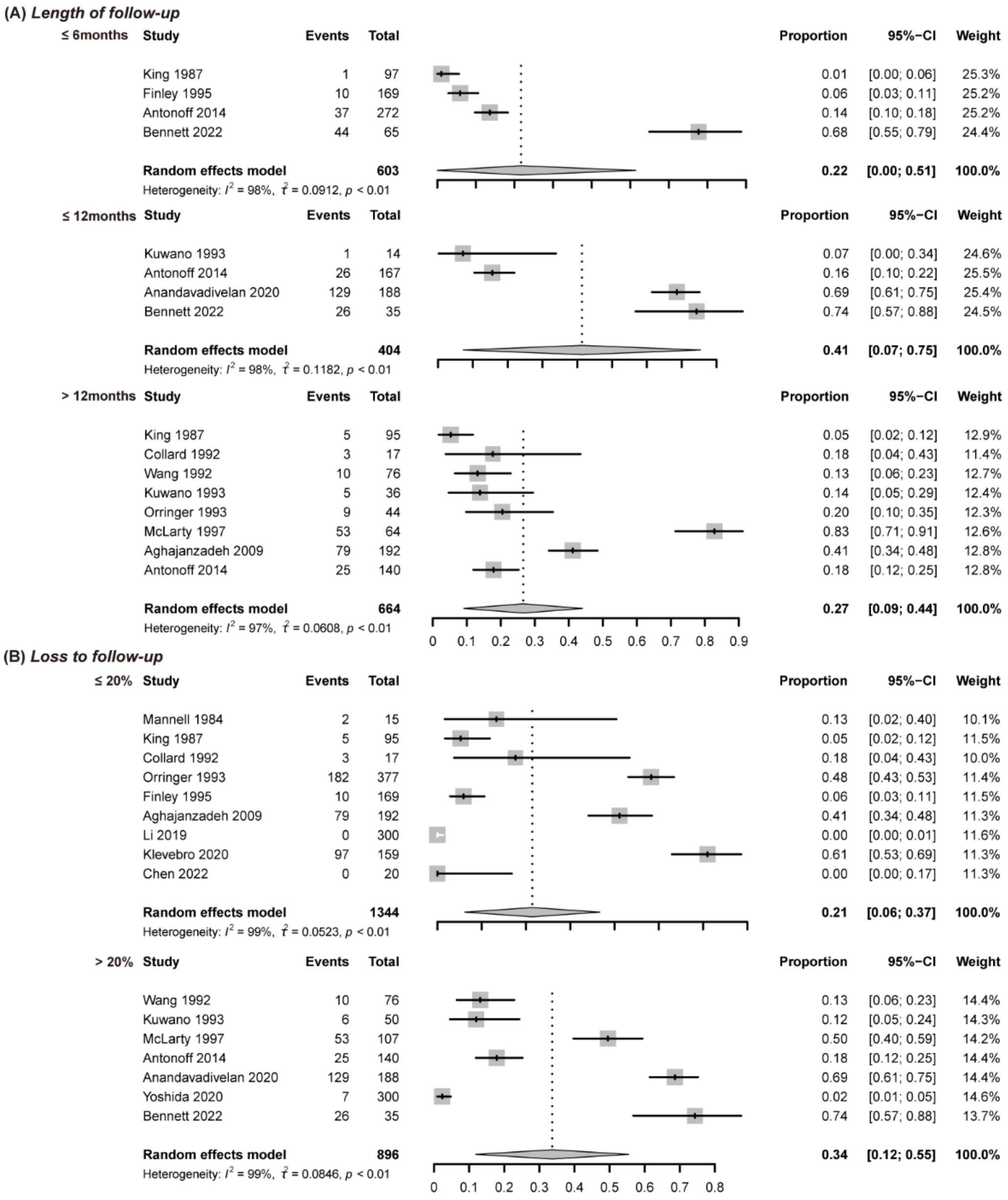

Supplementary Fig. 4 Forest plots for meta-analyses on the prevalence of dumping syndrome after esophageal cancer surgery by length (A) and completeness (B) of follow-up

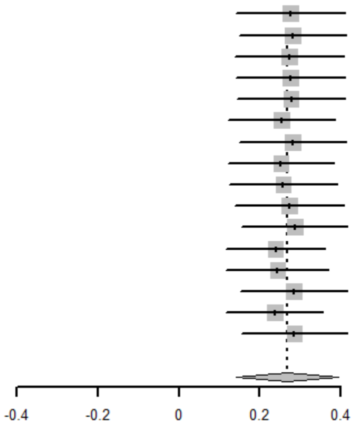

Study	Proportion	95%-CI	P-value	Tau2	Tau	I2
Omitting Mannell 1984	0.28	[0.14; 0.41]	.	0.0682	0.2611	99%
Omitting King 1987	0.28	[0.15; 0.41]	.	0.0661	0.2572	99%
Omitting Collard 1992	0.27	[0.14; 0.41]	.	0.0688	0.2622	99%
Omitting Wang 1992	0.28	[0.14; 0.41]	.	0.0683	0.2613	99%
Omitting Kuwano 1993	0.28	[0.14; 0.41]	.	0.0680	0.2608	99%
Omitting Orringer 1993	0.25	[0.12; 0.39]	.	0.0660	0.2570	99%
Omitting Finley 1995	0.28	[0.15; 0.41]	.	0.0663	0.2576	99%
Omitting McLarty 1997	0.25	[0.12; 0.38]	.	0.0657	0.2563	99%
Omitting Aghajanzadeh 2009	0.26	[0.12; 0.39]	.	0.0681	0.2609	99%
Omitting Antonoff 2014	0.27	[0.14; 0.41]	.	0.0691	0.2629	99%
Omitting Li 2019	0.29	[0.16; 0.42]	.	0.0640	0.2531	99%
Omitting Anandavadivelan 2020	0.24	[0.12; 0.36]	.	0.0558	0.2362	99%
Omitting Klevebro 2020	0.24	[0.12; 0.37]	.	0.0605	0.2460	99%
Omitting Yoshida 2020	0.28	[0.15; 0.42]	.	0.0650	0.2550	99%
Omitting Bennett 2022	0.24	[0.12; 0.36]	.	0.0541	0.2327	99%
Omitting Chen 2022	0.29	[0.16; 0.42]	.	0.0642	0.2533	99%
**Random effects model**	**0.27**	**[0.14; 0.39]**	**.**	**0.0649**	**0.2547**	**99%**

Supplementary Fig. 5 Sensitivity analysis by dropping one individual study at each time
